# Hancock Medical Association

**Published:** 1856-06

**Authors:** Geo. W. Hall

**Affiliations:** Secretary; Carthage, Ills.


### HANCOCK MEDICAL ASSOCIATION.

CARTHAGE, ILLS., April 16, 1856.

Met, Dr. Hooten presiding.

On motion the following officers were elected for the ensuing year:

President, Dr. Daniel Pierson; Vice President, Dr. David Ellis; Treasurer, Dr. Charles Hay; Secretary, Dr. Geo. W. Hall.

Professors McGugin and Marsh being present were elected honorary members, and requested to participate in the proceedings of the meeting. Professor McGugin exhibited a specimen of diseased heart accompanied with some highly interesting and instructive remarks on the same. His remarks were listened to with profound attention and on motion, he and his colleague were requested to hereafter meet with the Association. The retiring President, Dr. Hooten, delivered his valedictory address on the Philosophy of Remedial Action, in which the action of blisters and tartarized antimony were more particularly alluded to. Dr. W. S. Linn, who was present, was elected a member. An interesting paper was read by Dr. Ellis on Diagnosis. The Secretary elect read a paper on Medical Etiquette, a copy of which was on motion requested for publication in the Iowa Medical Journal. Several subjects connected with the practice of medicine were freely discussed in which most of those present took a part. The following gentlemen were appointed to prepare papers for the next meeting:

On Typhoid Fever, Dr. King; On Puerperal Fever, Dr. Linn; On Diarrhœa, Dr. Hooten; On Erysipelas, Dr. Coolidge; On Dysentery, Dr. Hall.

On motion the proceedings of the meeting were directed to be published in the Iowa Medical Journal and the Carthage Republican. The Association then adjourned to meet in Carthage on the 16th of July next.

GEO. W. HALL, *Secretary.*

